# Feminizing genital reconstruction in congenital adrenal hyperplasia

**DOI:** 10.4103/0970-1591.45532

**Published:** 2009

**Authors:** Jeffrey A. Leslie, Mark Patrick Cain, Richard Carlos Rink

**Affiliations:** Department of Urology, University of Texas Health Science, 7703 Floyd Curl Dr., MC 7845, San Antonio-78229, Texas - USA; 1 Indiana University, School of Medicine, Riley Hospital for Children, 702 Barnhill Drive #4230, Indianapolis-46202, Indiana - USA

**Keywords:** Congenital adrenal hyperplasia, feminizing genitoplasty, urogenital sinus, vaginoplasty

## Abstract

The past several decades have seen multiple advances in the surgical reconstruction for girls born with Disorders of Sexual Differentiation. This surgery can be technically very demanding, and must be individualized for each patient, as the degree of virilization and level of confluence of the vagina and urogenital sinus will dictate the surgical approach.

In this manuscript we present our approach and experience in the surgical options for girls born with Congenital Adrenal Hyperplasia, with special attention regarding clitoroplasty, urogenital mobilization, and vaginoplasty.

## INTRODUCTION

Few areas in pediatric urology are more challenging and controversial than genital reconstruction for girls with virilization. This situation is most commonly seen as a result of congenital adrenal hyperplasia (CAH). Debate exists mainly regarding the optimal timing and extent of genital reconstruction for these children. Additionally, the surgical reconstruction itself can be difficult, requiring meticulous attention to detail and tissue handling. The degree of virilization and level of the confluence of the vagina with the urogenital sinus is variable and unique to each girl. There are several options for surgical repair of these anomalies, which must be matched to the anatomy of the child to achieve a functional and cosmetic outcome. Knowledge of each of these techniques and their applicability is critical. The main focus of this review is to address the surgical aspects of feminizing genitoplasty for CAH, focusing on the different types of repairs in use today and when each should be employed.

## PREOPERATIVE CONSULTATION

As mentioned, both the timing and extent of surgery for girls with CAH is an area of intense debate. The key aspects of this debate should be explained to the family fully. A multi-disciplinary approach to these children is imperative, employing input from an endocrinologist, psychologist or psychiatrist, pediatric urologist/surgeon and most importantly, the family. We advocate a multi-disciplinary conference with each specialist and the family present, to discuss these issues together. It is common belief that all surgeons advocate early surgical reconstruction and all non-surgeons recommend observation and later surgery at the npatients’ own desire. It is our hope that none of these occurs. Rather, an open discussion of the issues at hand based on our current understanding of psychosexual development, current surgical techniques (as opposed to older techniques no longer employed), and family wishes should be the goal. We recommend providing the family with viewpoints on both ends of the spectrum. Parents should be encouraged to read and discuss the views held by national and international advocacy groups such as Congenital Adrenal Hyperplasia Research and Education Support (CARES) and the Intersex Society of North America (ISNA). Once the parents have had an opportunity to read and discuss these issues fully, they should be allowed to make an informed decision regarding the care of their child.

## PREOPERATIVE EVALUATION

For the purposes of this review, it is assumed that the diagnosis of CAH has been definitively made and endocrinologic evaluation and management has been established. It is also assumed that the multi-disciplinary conference and education mentioned previously has been completed and the parents have decided to proceed with early complete reconstruction. The techniques described herein, however, are applicable to those who defer reconstruction until the peri-pubertal time.

**Figure 1:**
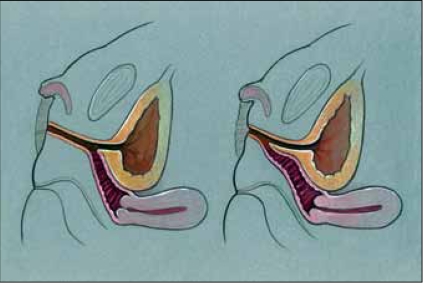
Urogenital sinus anatomy: vagina and urethra join and exit as common channel. High confluence (left), low confluence (right)

It is critical both pre- and post-operatively that endocrinologic control be maintained. Additionally, all children with CAH require stress-dose steroid administration peri-operatively, guided by an experienced pediatric endocrinologist. All children should undergo pelvic ultrasonography and genitography preoperatively. Occasionally, the bladder or vagina cannot be cannulated through the common urogenital sinus (UGS), requiring cystovaginoscopy to place catheters for genitography. If the surgeon is considerably experienced in all types of repair techniques, this can often be done initially under anesthesia at the time of planned repair. Otherwise, this should be performed under anesthesia as a purely diagnostic step. The goals of genitography are to opacify the bladder, vagina and the common UGS – especially the confluence of the vagina with the UGS – which is critical to determining the type of repair that should be chosen [Figure 1].

**Figure 2:**
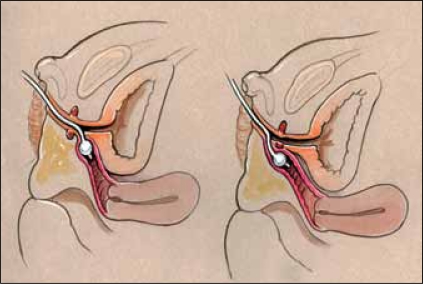
Fogarty catheter placed in the vagina in high confluence (left) and low confluence (right)

The reconstructive procedure should always begin with cystovaginoscopy performed by the surgeon doing the repair, to confirm the anatomy seen with genitography, verify the presence of a single cervix, and measure the location of the confluence relative to the introitus and more importantly, the bladder neck. We believe the latter is the most critical as this distance is important in determining the type of repair that should be performed, as well as defining the risks after the procedure.[1] After surveying the bladder and vagina, a small ureteral catheter can be introduced through the cystoscope into the vagina, to measure both the distance from the bladder neck to confluence and from confluence to the meatus of the UGS. This catheter can then be exchanged for a small Fogarty catheter, which is anchored in the vagina [Figure 2]. A Foley catheter is then advanced alongside the Fogarty into the bladder. The catheters are then secured to each other with a suture. These catheters should remain sterile, as they will be manipulated during the repair to follow.

**Figure 3:**
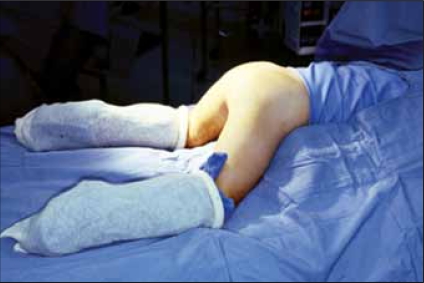
Total lower body preparation allows prone approach (From Rink RC, Adams MC: Feminizing Genitoplasty: State of the art. World J Urol 1998:16:212.)

The repair itself can be done in lithotomy position, but we prefer a “frog-legged” supine position. The child undergoes a circumferential prep from the nipples to toes and is then passed through the aperture in a laparotomy drape. The buttocks are elevated with sterile towels. This position has two distinct advantages over lithotomy position: 1) the child can be rotated to the prone position if needed, without the need to re-prep and risk contamination of the field [Figures 2 and 3] the exposure for assistants and observers is facilitated.[1]

A full bowel preparation or enemas should be given the day prior to surgery. We also irrigate the rectum “on the table” prior to beginning the repair, to minimize stool burden and rectal distention. Broad-spectrum intravenous antibiotics are administered prior to incision. If an epidural catheter is planned, we prefer to have this placed at the end of the repair, as the circumferential prep is compromised by the epidural catheter dressing.

## SURGICAL APPROACHES

There are three basic components to feminizing genitoplasty: clitoroplasty, labioplasty, and vaginoplasty. Debate still exists regarding when each of these components should be performed. Traditionally, vaginoplasty was deferred until at or after puberty, as revision for vaginal stenosis has been reportedly common when vaginoplasty is performed early. However, the advantage of performing vaginoplasty simultaneously with labioclitoroplasty is that the preputial skin is available to create labia minora and to possibly be used in the vaginoplasty. We prefer this approach and inform parents preoperatively that a minor revision at or after puberty may be necessary in some cases. When vaginoplasty is deferred initially, vaginoplasty is always required later anyway.

Thus, with either approach two procedures are usually required. We feel that the vaginoplasty is technically easier to perform early, usually requiring only a simple introitoplasty later, such as a Y-V advancement.

### Clitoroplasty

The degree of clitoral hypertrophy is quite variable in girls with CAH. Older techniques such as clitoral amputation and recession should not be used today. Clitoral recession can result in painful erections after puberty and newer techniques eliminate this problem. If possible, no clitoral surgery should be performed, as no evidence suggests that a large clitoris is detrimental to sexual function. However, in most cases, the clitoris is quite large, appearing more like a penis than a clitoris. In such cases, clitoral reduction should be discussed with the parents, based on knowledge of current techniques and neuroanatomy of the clitoris. Based on work by Baskin *et al.*,[2] the sensory nerves of the clitoris have been mapped and are analogous in location to the sensory nerves of the penis, with which pediatric urologic surgeons are quite familiar. These nerves course on the dorsal aspect of the clitoris, emanating from just under the pubis [Figure 4a]. Therefore, the dorsal aspect of the clitoris itself must not be disturbed. Kogan *et al.*,[3] originally described a sub-tunical excision of the erectile tissue which forms the basis of our current approach. As apposed to Kogan's lateral corporotomies, we employ ventral corporal incisions [Figure 4b]. With this technique, the dorsal neurovascular bundles on Buck's fascia are not manipulated, preserving innervation, and vascularity of the glans.

**Figure 4a:**
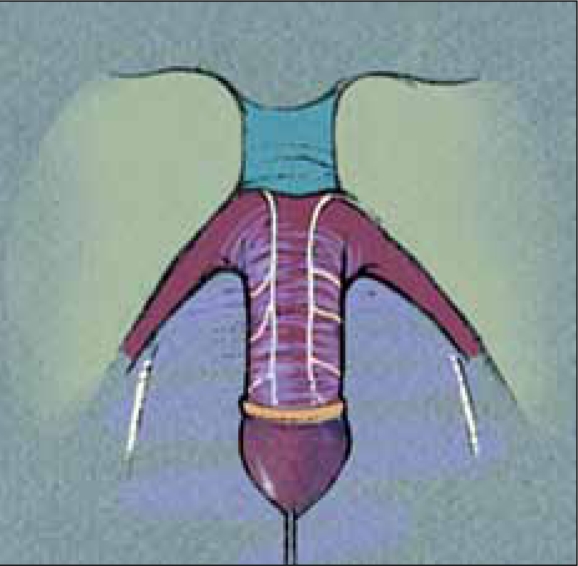
Hypertrophied clitoris with dorsal nerves and branches.

**Figure 4b:**
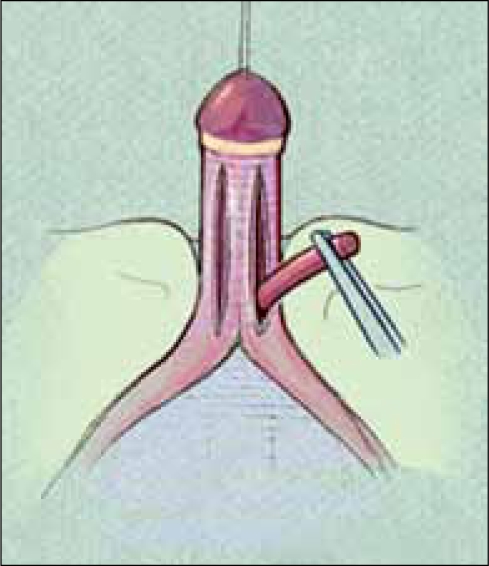
Hypertrophied clitoris with ventral corporotomies. Excision of spongy erectile tissue.

**Figure 5:**
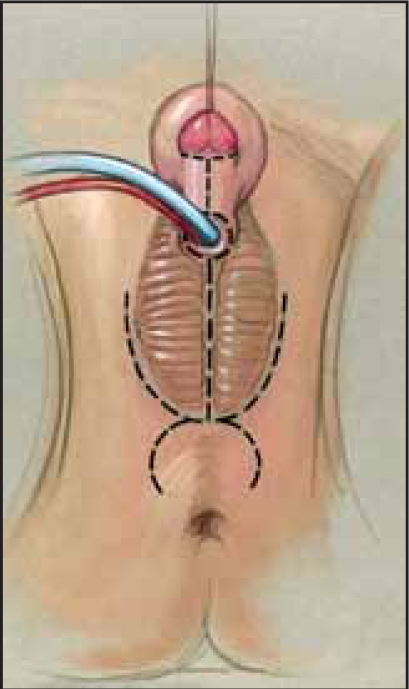
Proposed omega-shaped perineal skin flap

We generally perform clitoroplasty first, beginning with a traction suture in the glans. Skin lines are drawn parallel to the ventral urethral plate, around the UGS meatus, continuing in a near-circumferential manner around the dorsolateral aspects of the preputial skin. This is similar to the initial incision for hypospadias repairs, but leaving a larger dorsolateral “collar” of inner preputial/mucosal skin with the glans to allow for creation of a clitoral hood later. This skin has been shown to be second in sensitivity only to the glans itself.[4] The skin lines are infiltrated with 1:200,000 epinephrine for hemostasis. A posterior omega-shaped line is also outlined, anterior to the perineal body, with the apex of the incision located at the ultimate location of the vagina [Figure 5]. This location is best determined by palpating the ischial tuberosity and ensuring that the apex of the omega is just anterior to the tubersosity. The base of this flap should be kept wide and begins near the anus (the base of the omega will become the “perineal body”). The clitoris is then degloved, similar to hypospadias surgery, initially leaving the ventral strip of “urethral plate” (or extension of the UGS) intact. Once the dorsal skin is degloved, the distal portion of the UG sinus (the urethral plate) is divided transversely, just below the glans clitoris. This allows the distal mucosal strip and the proximal UGS to be mobilized off the ventral aspect of the corporal bodies to below the bifurcation of the corporal bodies. A suture can be placed in the distal UGS to provide traction during this dissection. Having now separated the UGS from the corpora, clitoral reduction can be performed. A tourniquet is placed around the corporal bodies at the bifurcation. Longitudinal ventral incisions in the tunica albuginea are made and the spongy erectile tissues are “teased” out of the corporal bodies quite easily. The proximal stumps of the erectile tissue are then suture-ligated, avoiding the dorsal tunics and their neurovascular bundles dorsally. The tourniquet is removed and hemostasis from the ligated stumps is ensured. The redundant dorsal tunica albuginea and neurovascular bundles can now be “folded” in half, suturing the body of the glans to the corporal body stumps with absorbable sutures. This should allow for painless engorgement of the proximal corpora with sexual activity. The folded tunics are secured in a subcutaneous pocket above the pubic bone by suturing the lateral tunical edges to the periostium of the pubis. The subcutaneous tissue and fat are closed in the midline to cover the neurovascular bundles and create a more normal superior mons pubis. The coronal collar is folded over the glans partially, creating a clitoral hood, sewing it to the anterior aspect of the labia minora later in the procedure during labioplasty.

The remainder of the preputial skin mobilized during de-gloving is divided in the midline and secured to the clitoral hood, as with a hypospadias repair. The right and left sides of the preputial skin are then rotated inferiorly and can be used to create labia minora. The medial edges are sewn to the exposed vagina on both sides.

### Labioplasty

**Figure 6:**
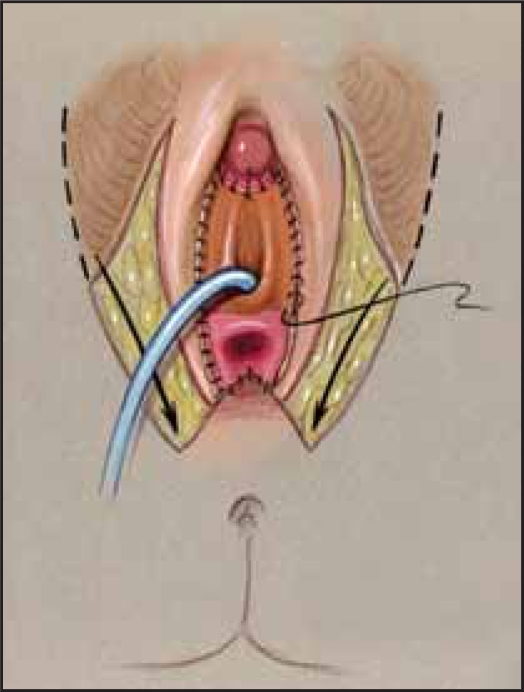
Labioplasty. Split phallic skin has been sewn in place as labia minora. Proposed Y-V plasty for labia majora.

Most girls with virilization from CAH have labioscrotal swellings which are more anterior than normal labia majora. Varying degrees of ruggation may be present, as well. To move this labioscrotal skin posteriorly, “Y” shaped incisions are made posterior to the swellings, with the confluence of the Y located at the posterior aspect of the swellings. After mobilizing these skin flaps, they can then be moved posteriorly, beside the introitus, performing bilateral Y-V-advancement. The medial aspects of these skin flaps will be sutured to the lateral edges of the preputial skin flaps mobilized during clitoroplasty (now labia minora) [Figure 6]. The end result is anatomically correct positioning of the labia minora and majora beside the introitus, rather than antero-laterally.

### Vaginoplasty

There are at least 4 basic types of vaginoplasty: cut-back, vaginal replacement (bowel/skin), posterior skin flap, and pull-through. Only the latter 2 of these are usually appropriate for reconstruction for girls with CAH. Vaginal replacement is usually only needed for vaginal atresia or agenesis, which does not occur with CAH. A cut-back vaginoplasty is only indicated for simple labial fusion. The majority of girls with CAH can be managed with a flap vaginoplasty, which is indicated for the low and moderate confluence UGS. Flap vaginoplasty should not be used alone for a high confluence, as it does nothing to move the confluence closer to the perineum, and leaves the urethra in a severely hypospadiac position. For the high confluence, a pull-through vaginoplasty may be required. Urogenital mobilization can also be readily combined with these techniques and will be discussed later.

### Vaginoplasty for low confluence UGS (Flap)

As mentioned, a flap vaginoplasty is usually adequate for the low confluence and many moderate level UGS anomalies, commonly seen in girls with CAH. Once the omega-based flap has been created and the UGS mobilized back to the bifurcation of the corporal bodies, further dissection posteriorly, along the ventral surface of the UGS will usually allow the Fogarty balloon in the vagina to be palpated. In the classic flap vaginoplasty, once this area of the UGS has been adequately mobilized, the UGS is opened ventrally, exposing the posterior wall of the vagina and the confluence of the UGS. With urogenital mobilization techniques, before spatulating the UGS either on the ventral or dorsal aspect, a decision should be made regarding how the UGS mucosal tissue will be utilized (described later). The posterior wall of the mobilized vagina is spatulated in the midline until normal caliber vagina is encountered. It is critical that the surgeon ensure that normal caliber vagina has been reached; otherwise, stenosis is likely to result. The perineally-based omega skin flap is then anastomosed to the spatulated posterior vaginal wall with interrupted sutures [Figure 7a, b].

**Figure 7a:**
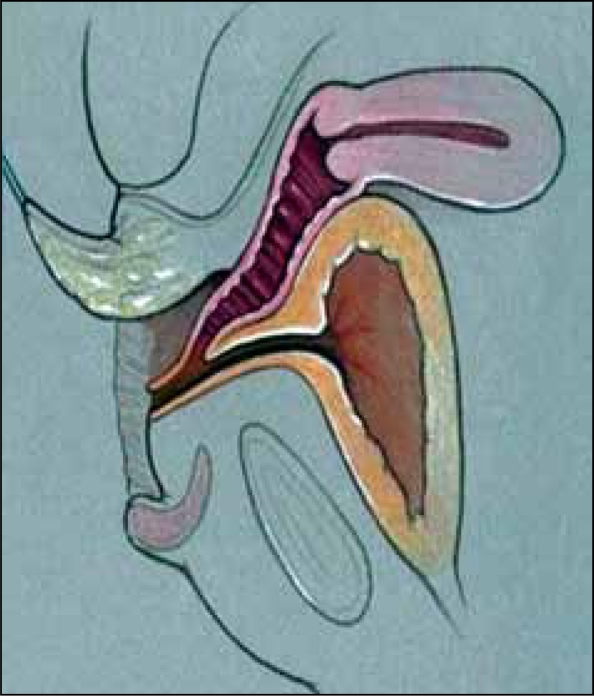
Flap vaginoplasty a. Perineal flap mobilized

**Figure 7b:**
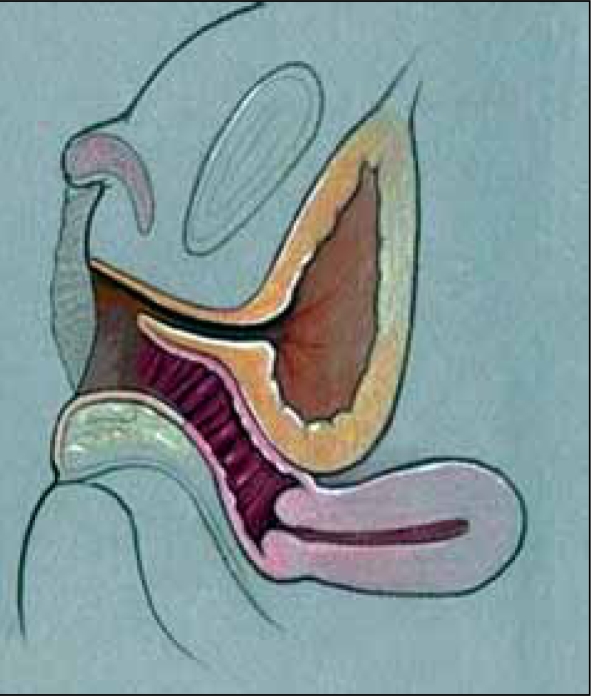
Flap vaginoplasty b. Flap sewn to opened sinus and vagina.

### Vaginoplasty for moderate or high confluence UGS (Pull-through)

Since a flap vaginoplasty alone does not bring the confluence of the vagina with the UGS any closer to the perineum, it is not suitable for higher confluence repairs as a sole technique. Historically, the majority of girls with a high confluence will require separation of the vagina from the UGS with a pull-through vaginoplasty, initially described by Hendren and Crawford.[5] The initial portions of the dissection are similar to the description previously mentioned for lower confluences. In higher confluences, though, the Fogarty balloon may not be palpable or may be too high to obtain adequate exposure to divide the vagina off of the sinus and perform urethroplasty. In such cases, we have found rotating to the prone position to be very helpful with visualization and exposure [Figure 8a]. Once the posterior wall has been opened in the midline as described above, the anterior wall of the vagina must be separated from the urinary tract in a pull-through procedure [Figure 8b]. The opening in the sinus is then closed in layers with absorbable suture [Figure 8c]. The UGS will become the urethra. The vagina is then mobilized circumferentially towards the perineum and anastomosed to the perineally based skin flap as described for flap vaginoplasty. In the classic Hendren pull-through, the anterior vaginal wall was created from skin flaps. With the advent of urogenital mobilization, the redundant UGS sinus tissue can easily be split dorsally and used as a Passerini flap to create the anterior wall [Figure 9a,b].

**Figure 8a:**
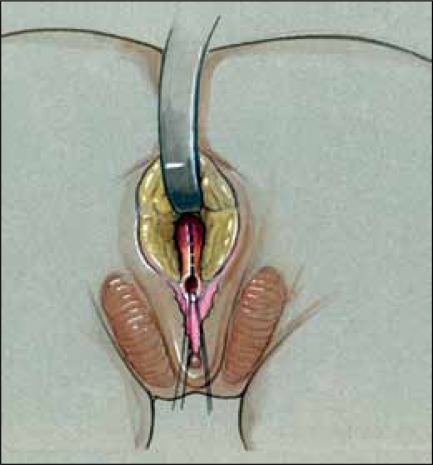
Pull-through vaginoplasty. a. Prone approach, exposing confluence with proposed midline incision to open sinus and vagina.

**Figure 8b:**
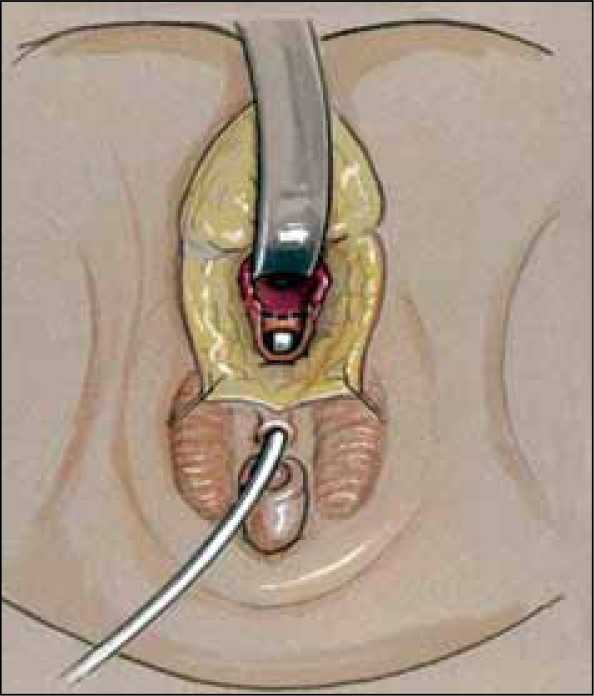
Retractor in vagina with proposed incision to separate vagina from urethra.

**Figure 8c:**
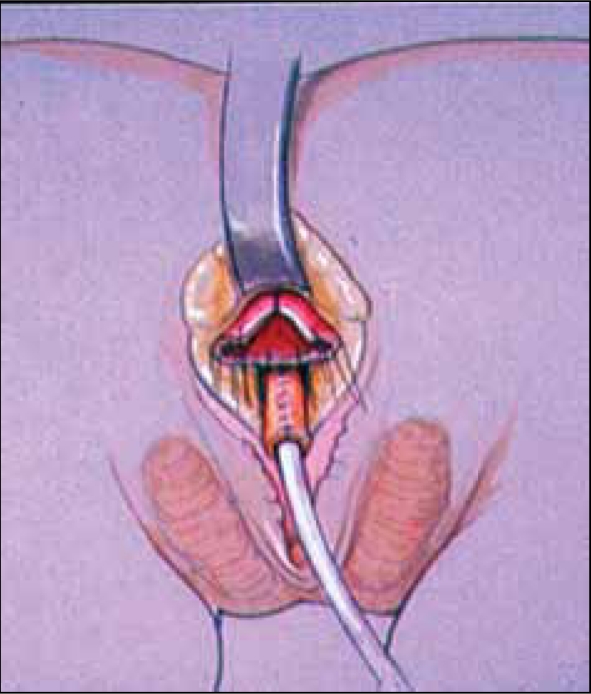
Vagina mobilized and posterior vaginal wall spatulated. Sinus tubularized to create urethra.

**Figure 9:**
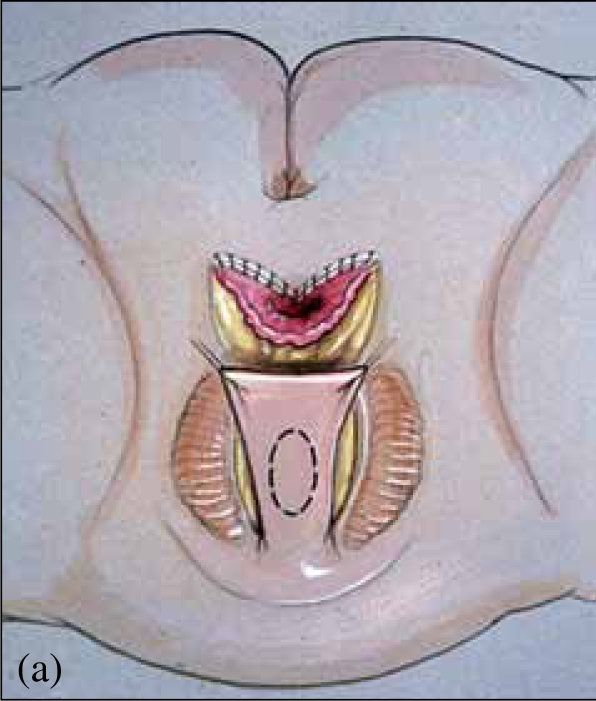

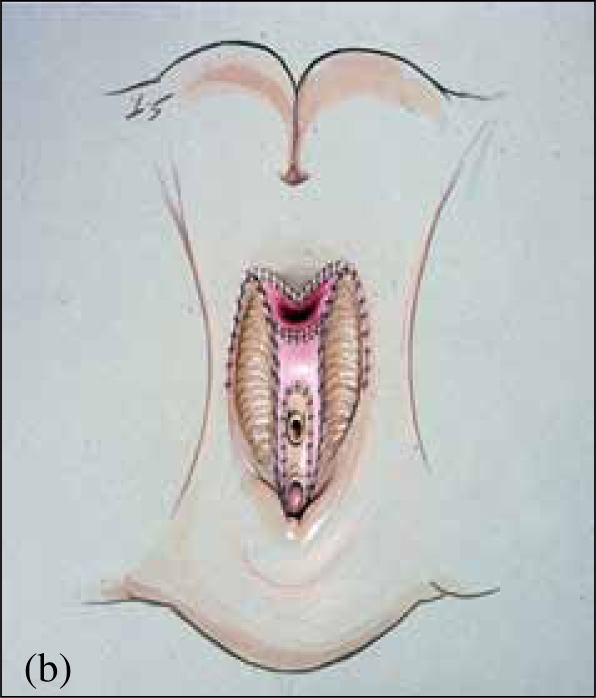
(a) Modified Gonzalez preputial flap to create an anterior vaginal wall; the posterior flap has been anastomosed to the spatulated vagina (b) Creation of mucosa-lined vestibule and Y-V-labioplasty

### Urogenital mobilization

Total urogenital mobilization (TUM) was described in 1997 by Alberto Pena as a means to repair the UGS component of a cloaca.[6] Its use has since been expanded to include most UGS repairs today. In TUM, the urogenital sinus is dissected circumferentially, including anteriorly, as a single unit [Figure 10a]. This allows the confluence of the vagina to be brought closer to the perineum, minimizing the extent of skin flaps necessary for lower confluences and improving exposure for pull-through procedures. The posterior dissection is similar to that done for a pull-through or flap procedure, with careful mobilization of the UGS off of the rectum, staying in the midline at all times for the initial dissection. The anterior dissection in TUM extends beyond the pubourethral ligament, under the pubis, providing significant mobilization of the sinus [Figure 10b].

**Figure 10a:**
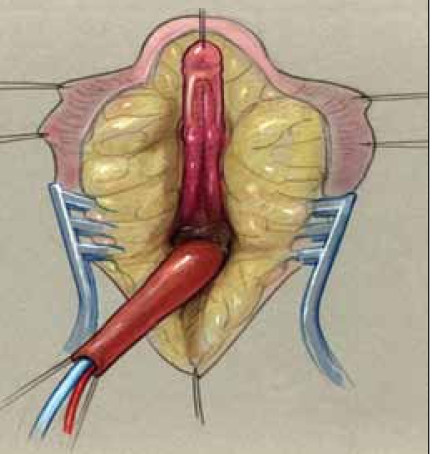
Total urogenital mobilization. a. AP view with the sinus mobilized.

**Figure 10b:**
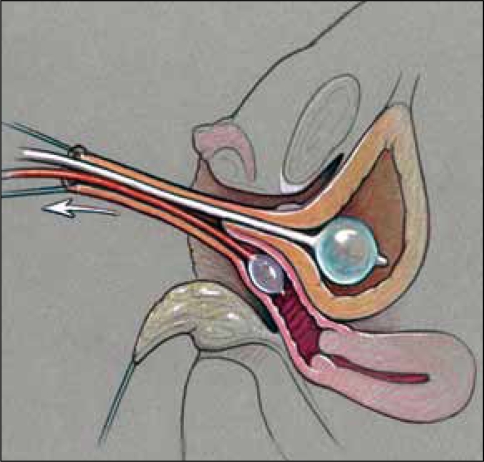
The sinus is mobilized circumferentially, including beneath the pubis.

**Figure 11:**
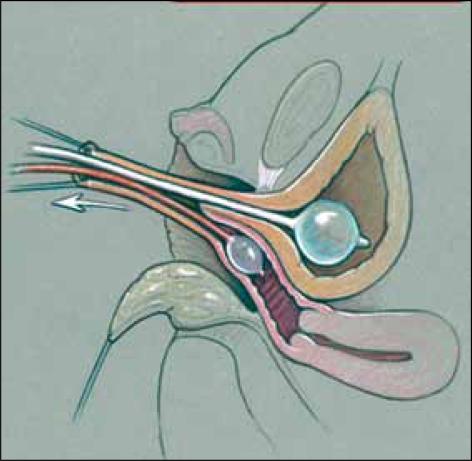
Partial urogenital mobilization. Dissection stops at the pubourethral ligament anteriorly.

Due to concerns for potential urinary incontinence problems from the circumferential dissection of the UGS proximal to the pubourethral ligament, Rink *et al*. proposed partial urogenital mobilization (PUM).[7] In PUM, the dissection anteriorly ceases at the pubourethral ligament [Figure 11]. In all but the most extreme cases, this is adequate for repairs in girls with CAH. If further mobilization is needed, however, the dissection can be easily continued under the pubis, converting to TUM.

In our experience, urogenital mobilization greatly facilitates the repair, regardless of whether a pull-through or flap vaginoplasty is being performed. By circumferentially mobilizing the entire UGS, the vagina is moved closer to the perineal skin, minimizing the extent of skin flaps required and in some cases allowing a flap vaginoplasty to be performed rather than a pull-through, with its inherent requirement to divide the vagina off the UGS. We now routinely employ PUM in all our repairs and have been pleased with the outcomes.

**Figure 12:**
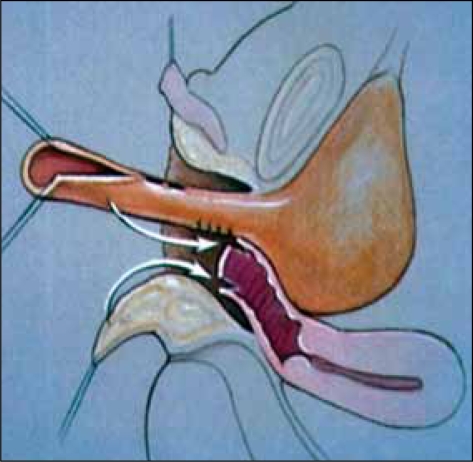
The sinus can be split dorsally to create an anterior vaginal wall for pull-through vaginoplasty as a Passerini flap

We have also described three ways that the redundant UGS tissue can be used for various purposes for either tissue coverage of the introitus or to augment the vagina rather than use skin flaps.[7,8] This tissue is available with either PUM or TUM. Once the UGS has been adequately mobilized, the UGS is spatulated. This can be done anteriorly, dorsally, or laterally. This decision should be based on the vaginal location, the type of repair, and the tissue requirements. In a pull-through procedure, the dorsal side of the UGS is spatulated and the anterior wall of the vagina can be augmented with the redundant tissue, as in a Passerini flap repair [Figure 12]. In a flap vaginoplasty, the ventral side of the UGS is opened, and the redundant tissue can be used to create a mucosa-lined vestibule [Figure 13a–c]. Lastly, in a low to mid-level confluence, lateral division of the sinus allows the redundant sinus to be spiraled around and placed into the spatulated vagina to create a posterior vaginal flap, rather than using the skin omega flap [Figure 14a–d].

**Figure 13a:**
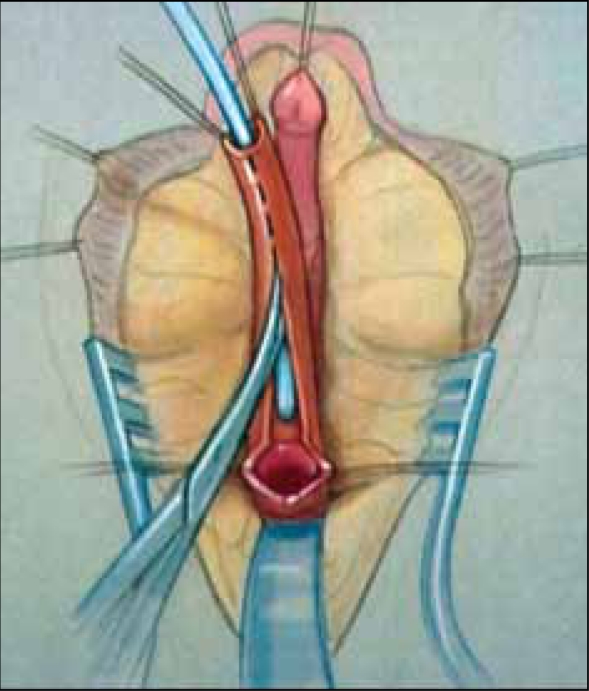
Ventral incision in the mobilized sinus.

**Figure 13b:**
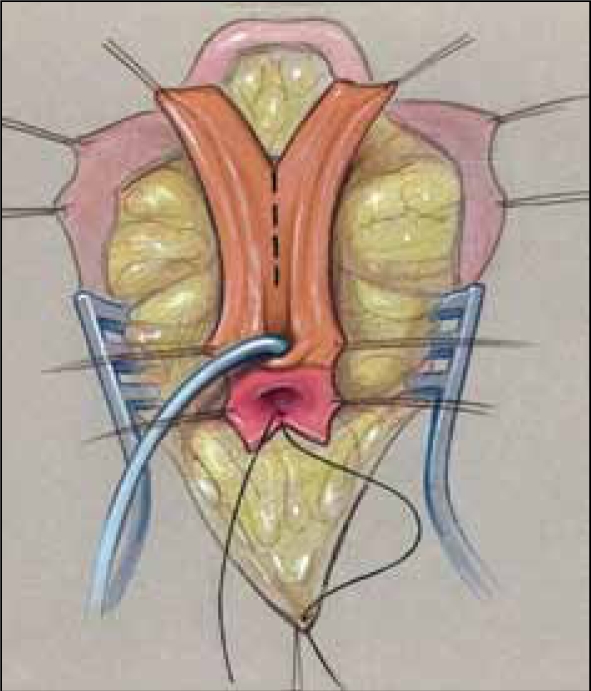
Sinus partially opened dorsally.

**Figure 13c:**
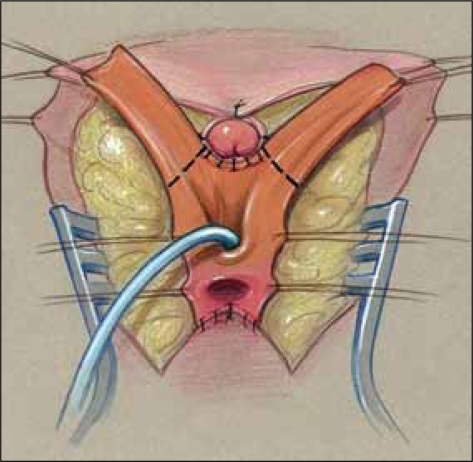
Creation of a mucosa-lined vestibule.

**Figure 14a:**
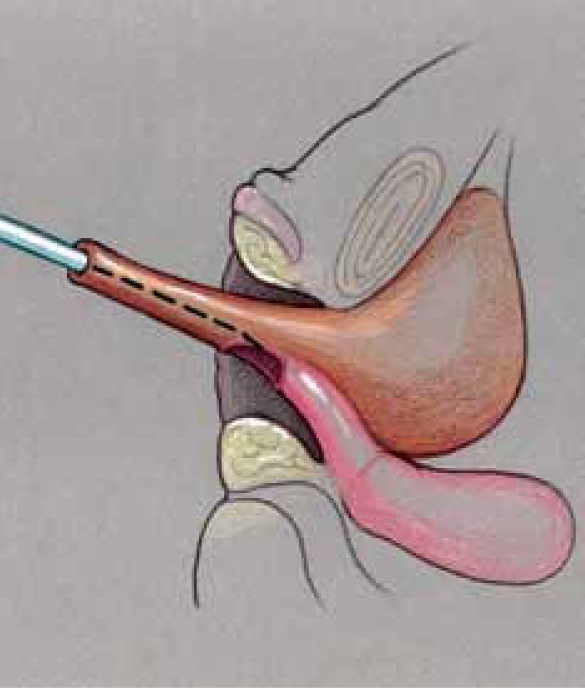
Proposed lateral incision in mobilized sinus.

**Figure 14b:**
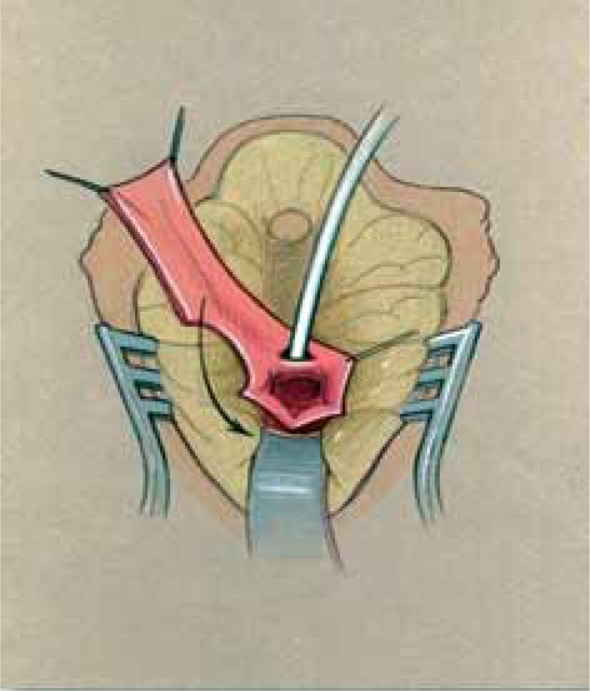
Mobilized sinus after lateral incision.

**Figure 14c:**
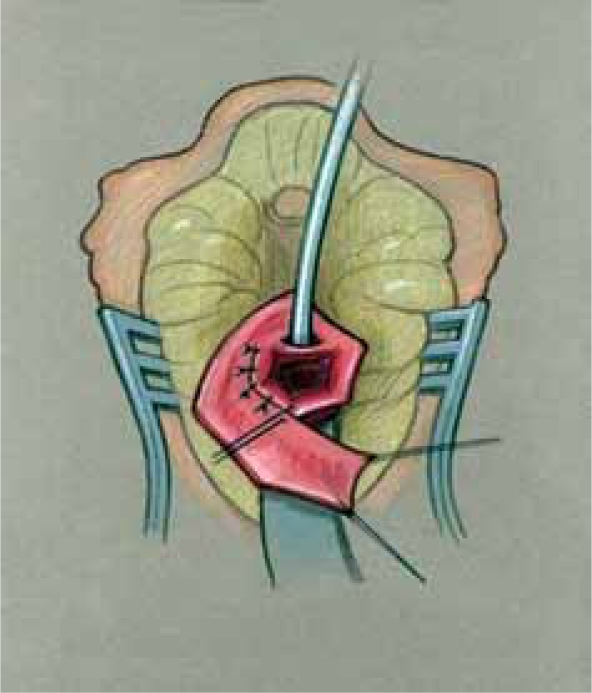
Sinus tissue spiraled around vagina to create mucosal lined vestibule.

**Figure 14d:**
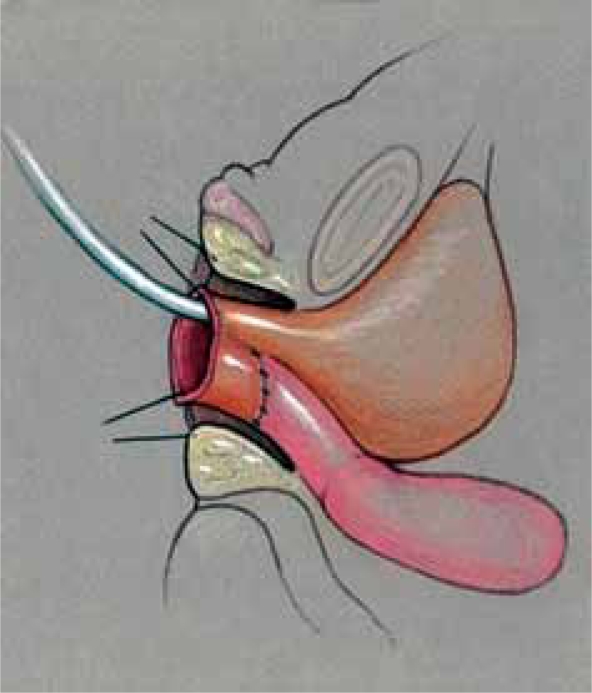
Lateral view at completion.

As for flap repairs, the posterior wall of the mobilized vagina is then spatulated in the ventral midline, until normal caliber vagina is encountered. The perineally-based omega skin flap is then anastomosed to the spatulated posterior vaginal wall with interrupted sutures.

## POST-OPERATIVE CARE

At the end of the repair, we place a Penrose drain in the vagina, securing it to the perineal skin with a suture. This is removed prior to discharge from the hospital. A Foley catheter is used for bladder drainage for several days. A compressive perineal dressing is placed, secured to the buttocks and groin with tape. The legs are then “wrapped” together from the hips to the ankles with an ACE bandage, with foam padding placed between the knees. This “mermaid wrap” is left on for 2-3 days post-operatively, to minimize movement and abduction of the thighs. An epidural catheter can be placed by the anesthesiologist and is very helpful with post-operative analgesia in our experience. The child is kept fasting for 24 hours post-operatively, and then advanced from clear liquids to an age appropriate diet. The dressing is removed prior to or with the first bowel movement. Broad spectrum antibiotics are continued for 24-48 h post-operatively.

## OUTCOMES

### Clitoroplasty

Much of the debate about female genital reconstruction centers on very appropriate concerns for potential irreversible loss of clitoral sensation and poor cosmesis. Obviously, older techniques such as clitorectomy or clitoral recession are to be condemned. However, advances in the understanding of the anatomy of the clitoris and innervation of the glans have led to new techniques which preserve clitoral sensation and result in anatomically correct and cosmetic outcomes. Unfortunately, most reports on outcomes from clitoroplasty include a mixture of patients who underwent a variety of different procedures, including the older techniques which are no longer used.[9–14] Outcomes from these reviews, concerning both cosmesis and function, are quite variable as well. Long-term results from patients who have undergone modern clitoroplasty are lacking to date, as these techniques are relatively new and few have reached sexual maturity. However, short-term results are promising both cosmetically and functionally.[15,16]

### Vaginoplasty

As with clitoroplasty, outcomes from vaginoplasty should consider function as well as cosmesis. In addition to allowing tampon insertion and egress of menstrual fluid, the reconstructed vagina should allow for painless intercourse with adequate lubrication. While the need for vaginoplasty itself is not as controversial as clitoral surgery, the timing of vaginoplasty is a point of debate. The crux of this debate is centered on the high incidence of revisional surgery needed at or after puberty for most girls who undergo early or infant vaginoplasty. The most common problem requiring revision is vaginal or introital stenosis. In our experience, and others, this complication is often the result of failure to exteriorize the normal caliber vagina adequately or due to scar formation.[17] The majority of series in the literature describing follow-up after early vaginoplasty report high re-operation rates at or after puberty.[9,13,14,18–23] We freely acknowledge this to the families of our patients. However, many of these same authors recommend early vaginoplasty, as the revisional surgery needed around puberty is usually relatively minor, with excellent long-term results.[14,19,20]

Our preference is for early vaginoplasty in all but the most severe cases in which the vagina is very small and high. In such cases, we defer vaginoplasty until puberty, performing only a clitorolabioplasty in infancy, allowing the vagina to dilate after menarche in most cases. It should be noted as well, that we do not advocate vaginal dilation in prepubertal girls.

### Urogenital mobilization

We have recently reviewed our series of children who have undergone feminizing genitoplasty incorporating either TUM or PUM, focusing on urinary continence outcomes. Eighteen children underwent TUM, of whom only seven are neurologically normal. Median age at surgery for the TUM group was 13 months (3-174 months), with median follow-up of 33 months. Twenty-six underwent PUM, of whom 25 are neurologically normal. Median age of surgery for the PUM group was 12 months (4-149 months), with median follow-up of 28 months. Including both groups, neurologically normal children >3 years old are all continent. One child does have vaginal stenosis on early follow-up and cosmetics have been excellent. Thus, while it appears that TUM may be safe from an early continence standpoint, there are many unknowns with this procedure and we believe PUM is inherently safer. Currently, we prefer stopping the anterior dissection at the pubourethral ligament whenever possible and reserve the TUM for only the very high vaginal confluence.

## CONCLUSIONS

Management and surgical reconstruction for girls with congenital adrenal hyperplasia (CAH) remains a controversial and emotion-laden area in pediatric urology. Full disclosure of all of the relevant issues regarding timing and extent of surgery must be related to the family of these children, allowing the families to make truly informed decisions. The surgeon must be cognizant of the anatomy of each child and which repairs are therefore appropriate. Most importantly, long-term follow-up (after initiation of sexual activity) of current techniques is desperately needed to continue to provide these patients with not only good cosmetic outcomes, but also good functional outcomes.
